# The Use of a Suture Bridge Technique for Arthroscopic Rotator Cuff Repair in Patients Under 40 Years of Age Resulted in Successful Tendon Healing, Pain Relief, Improved Shoulder Function, and High Patient Satisfaction at a Minimum of 5-Year Follow-Up

**DOI:** 10.1016/j.asmr.2024.101009

**Published:** 2024-09-26

**Authors:** Laurentiu Cosmin Focsa, Faisal Adi, Marc-Antoine Rousseau, Patrick Boyer

**Affiliations:** Orthopedics and Trauma Surgery Department, Centre Hospitalier Universitaire Bichat-Beaujon, Assistance Publique - Hôpitaux de Paris, Paris Cité University, Paris, France

## Abstract

**Purpose:**

To evaluate the long-term clinical and imaging outcomes after arthroscopic repair of rotator cuff tears (RCTs) using a suture bridge technique in patients under 40 years of age.

**Methods:**

We retrospectively identified a consecutive series of patients who were treated arthroscopically for RCTs by a single surgeon between 2016 and 2018. Both full-thickness tears and partial tears were included. Arthroscopic cuff repair was performed with a double-row suture bridge technique using braided suture tapes. To assess clinical outcomes, we used the Constant score, the American Shoulder and Elbow Surgeons (ASES) score, a strength score, and a visual analog scale score. Tendon healing was evaluated at 12 months on magnetic resonance imaging using the Sugaya classification.

**Results:**

A total of 63 patients were included in the study. The mean age at the time of surgery was 33.6 years (range, 21-40 years), and the minimum follow-up duration was 5 years. Of the patients, 39 reported occasional sports activities and 8 were professional athletes whereas 16 reported no sportive activity prior to first symptoms. The mean follow-up duration was 66.8 months (range, 62.4-88.6 months). A significant improvement was observed in both the Constant score and the ASES score. The mean Constant score increased significantly from 39.8 points (range, 29-52 points) to 88.9 points (range, 34-100) postoperatively (*P* < .001). Similarly, the ASES score improved significantly from 41.8 points (range, 30-64 points) to 90.2 points (range, 35-100 points; *P* < .001). Mean anterior flexion improved from 86° (range, 60°-110°) to 137° (range, 90°-180°; *P* < .001). Pain significantly decreased after surgery, with the visual analog scale score descending from 6.3 (range, 3-10) to 1.3 (range, 0-9) postoperatively. The overall rate of return to previous activities in the cohort was 84% at an average of 10.1 months (range, 6-12 months) after surgery. Among the included patients, 85% who engaged in occasional sports activities and 67% of elite athletes returned to their preinjury sports levels after 9.8 months (range, 6-12 months) and 10.7 months (range, 6-12 months), respectively. Cuff rerupture occurred in 2 patients (3%), and tendon nonhealing (Sugaya stage 3 or 4) was observed in 5 patients (7%). At final follow-up, 95% of patients were satisfied or very satisfied with their functional results.

**Conclusions:**

The use of a suture bridge technique in arthroscopic RCT repair for patients under 40 years of age resulted in excellent long-term outcomes, including successful tendon healing, pain relief, improved shoulder function, and high patient satisfaction. This result is of significant importance in this demanding population because it allows satisfactory high rate of return to the preinjury level of activity.

**Level of Evidence:**

Level IV, therapeutic case series.

Whereas rotator cuff tears (RCTs) are well documented in older individuals and extensively studied, they are less commonly observed in the younger population, particularly those under 40 years of age.^1^ In the general population, the prevalence of RCTs was estimated at around 20.7%, but this may differ depending on the age distribution and primary industries within a community.^2^ In patients aged 20 to 50 years, the reported prevalence was 5.1%.^3^ Appropriate diagnosis and treatment of RCT are paramount, especially in young patients, given the critical need to maintain long-lasting structural and functional integrity of the shoulder. Arthroscopic repair represents the gold standard for RCT, resulting in a low incidence of complications, satisfactory recovery of mobility, effective pain relief, and restoration of shoulder biomechanics, avoiding ascension of the humeral head.^4^^,^^5^ Partial-thickness tears can be treated by either completion and repair or in situ transtendon repair because both strategies yield similar postoperative outcomes.^6^ Repairing supraspinatus tears can reverse increased glenohumeral loads. Restoring the native contact area and pressure within the glenohumeral joint can significantly reduce joint loads and ensure stable joint kinematics, potentially preventing the development of degenerative changes.^7^

Surgery aims to reattach the ruptured tendon to its anatomic footprint to aid healing and restore rotator cuff biomechanics. This prevents tendon retraction, muscular atrophy, fatty infiltration, humeral head migration, and arthritis, resulting in better mobility and pain relief. The degenerative process is also observed in incomplete RCT tears, with the risk of fatty infiltration increasing with the size of the rupture and the number of tendons involved.^8^^,^^9^

Two of the various techniques described for arthroscopic RCT repair are single-row and double-row techniques. Among the double-row methods, a prevalent approach involves anchor interconnection, commonly referred to as “suture bridge repair.” Clinical outcomes documented in the literature have shown comparability between these 2 techniques. However, double-row repair has shown a reduced incidence of revision and improved tendon healing.^10^, ^11^, ^12^ Dual-row repairs have shown their efficacy by obtaining a large insertion area, higher load to failure, and higher radiologic healing rate with long-term follow-up.^13^ Suture bridge repair outperforms conventional double-row techniques biomechanically, offering better load resistance, increased pressure at the bone-tendon interface, and wider coverage. The use of suture tapes distributes constraints broadly, reducing the risk of tendon tearing.^11^^,^^14^, ^15^, ^16^ The results of rotator cuff repair in the elderly population have been well studied in the literature, contrary to the results obtained in the younger population, for whom we find a lack of studies.^17^ In patients older than 65 years, the reported mean Constant score was 78 points, with patients presenting anterior flexion around 175°.^18^ Young patients reported a low satisfaction rate after surgery (68%) compared with older patients.^19^ This population has specifically higher functional, professional, and athletic demands compared with older subjects, which also makes this population more prone to retears.^20^

The purpose of this study was to evaluate the long-term clinical and imaging outcomes after arthroscopic repair of RCTs using a suture bridge technique in patients under 40 years of age. We hypothesized that the present reparation technique could improve both functional and imaging outcomes while also leading to a low failure rate.

## Methods

### Patients

Patients who underwent RCT repair between 2016 and 2018 performed by a single surgeon (P.B.) were identified for inclusion. All included patients had either a full-thickness or partial tear of the rotator cuff tendons confirmed intraoperatively.

The inclusion criteria were patients under 40 years of age at the time of surgery presenting with full-thickness or incomplete RCT, fatty infiltration grade less than 2 according to the Goutallier-Bernageau classification, and minimum clinical follow-up period of 5 years with magnetic resonance imaging (MRI) at 12 months. Biceps tenotomy-tenodesis and subacromial decompression were associated procedures with rotator cuff repair in our cohort. The exclusion criteria included single-row technique, shoulder stiffness, history of shoulder dislocation, proximal nonreducible retraction of tendons and/or association with superior migration of the humeral head, and osteoarthritis greater than grade 2 per the Hamada classification.

A preoperative interscalene brachial plexus block was administered using a combination of 0.5% ropivacaine and 0.5% dexamethasone, in addition to general anesthesia. In the postoperative period, a multimodal analgesia protocol was implemented for all patients, incorporating painkillers, ice, and immobilization.

### Surgical Procedure

**Fig 1:**
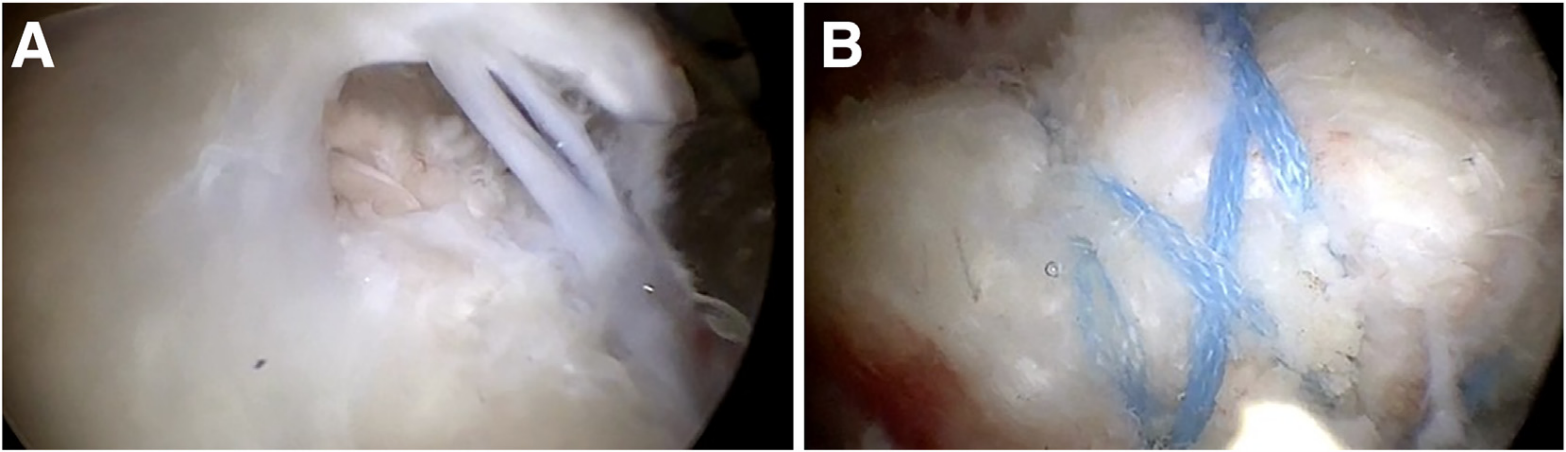
Arthroscopic view of rupture and repair. (A) Large full-thickness torn supraspinatus tendon in right shoulder, with scope in posterolateral portal. (B) Repair of full-thickness rupture of supraspinatus tendon in right shoulder using suture bridge system with medial knots, with scope in posterolateral portal.

The patients were positioned in the beach-chair position before surgery, following a step-by-step protocol to ensure security and mitigate complications associated with patient positioning. The surgical procedure commenced with articular exploration through the standard posterior viewing portal. Subsequently, extra-articular exploration in the subacromial space was conducted. After debridement and footprint preparation, reducibility was assessed. The primary suture technique used for repair involved a suture bridge system. Suture anchors (SwiveLock, 4.75 mm; Arthrex, Naples, FL) charged with 1 suture tape each (2-mm FiberTape; Arthrex) were positioned at the articular margin of the humeral head, forming the medial row. Subsequently, each anchor’s 2 strands were threaded through the tendon, approximately 5 mm lateral to the muscle-tendon junction of the rotator cuff, by a mattress technique. Traversing strands allowed tightening of the first row of anchors and application of the tendon to the bone. A connection between the anchors was established through strands that crossed in the lateral row (Fig 1). If necessary, the technique was completed by a simple stitch. After the final evaluation of the repair, acromioplasty was performed in patients presenting with subacromial impingement localized between the superficial surface of the supraspinatus and the subacromial bursa. Subacromial decompression was conducted through the lateral portal and completed through the posterior portal. Biceps tenotomy was carried out in patients with intra-articular lesions of the long head based on factors such as sex, age, and manual activity. Suprapectoral tenodesis was systematically performed using a lasso loop (FiberLoop; Arthrex) combined with anchor fixation at the intra-articular extremity of the biceps groove.

### Postoperative Protocol

Postoperatively, patients underwent 3 weeks of immobilization in a shoulder sling. During this period, patients were authorized to engage in passive mobilization, such as pendular movements of the upper limb, facilitated by a physiotherapist. Active motion initiation occurred at 3 weeks after surgery, whereas rotator cuff–strengthening exercises commenced between 10 and 12 weeks after surgery. Full return to sports and heavy labor activities was permitted after 6 months, contingent on individual functional recovery.^21^

### Functional Assessment

Primary outcomes included the mean Constant score, range of motion (measured with a goniometer), and strength evaluated both preoperatively and at latest follow-up. Abductor muscle strength was quantified by assigning 2 points for each kilogram maintained in isometric contraction in the horizontal plane and at 90° of abduction, with a maximum attainable score of 25 points.^22^ Secondary outcomes encompassed assessment of the American Shoulder and Elbow Surgeons (ASES) score, pain measured using a visual analog scale (VAS), and patient satisfaction assessed on an ordinal scale. The minimal clinically important difference (MCID) value was calculated using the following formula: one-half of standard deviation (SD) of delta (Δ) in score.^23^ All complications, such as shoulder stiffness, sepsis, complex regional pain syndrome, and retear, were recorded. Clinical outcomes were consistently evaluated at regular intervals by an impartial observer, a senior orthopaedic surgeon (L.C.F.), who maintained complete detachment from the surgical treatment of the patients throughout both the preoperative and postoperative phases.

### Imaging Assessment

Before surgery, profile and anteroposterior radiographs in neutral, internal, and external rotation were obtained in all patients to evaluate for possible calcifications of the rotator cuff, perform osteoarthritis grading, and assess superior migration of the humeral head. MRI was performed in the preoperative period using T1 and T2 fat-supression frontal, coronal, and transversal acquisitions in all patients.

**Fig 2:**
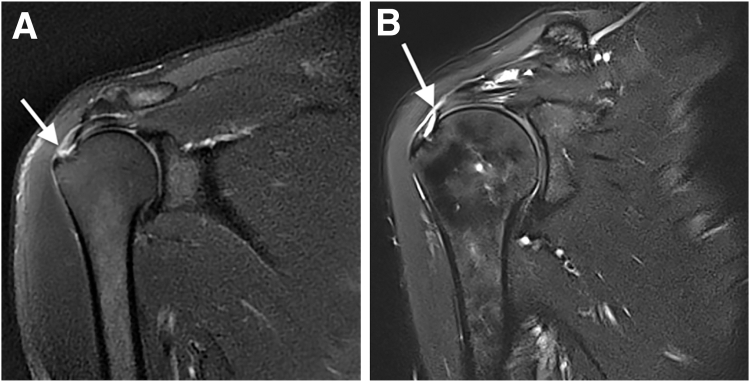
Magnetic resonance imaging (T2 fat-supression frontal view) of right shoulder showing full-thickness tear of supraspinatus tendon (arrow) presenting with distal retraction (Patte type 1) (A) and superficial partial tear of supraspinatus tendon (arrow) (B).

An RCT was defined as a complete or incomplete discontinuity of the tendon presenting as a hypersignal on T2 acquisition (Fig 2). Retraction due to a full-thickness RCT was staged using the Patte classification.^24^ In Patte type 1, the proximal stump is located near the bony insertion; in type 2, the proximal stump is at the level of the humeral head; and in type 3, the proximal stump is at the level of the glenoid or more proximal.

Furthermore, preoperative MRI scans were used to evaluate fatty infiltration and muscle atrophy based on the Goutallier classification, ensuring that the criteria for inclusion in the study were met. MRI of the operated shoulder was routinely performed at 12 months after surgery as part of normal clinical practice to assess tendon healing.

The Sugaya classification was used to assess tendon healing and was reported in our study as a primary outcome. Thus, Sugaya stage 1 and 2 tendons were considered healed whereas stages 3, 4, and 5 were considered failures in this study. A complete repair was defined as reconstruction of the anatomic footprint.

In patients presenting with clinical signs of adhesive capsulitis (active and passive range-of-motion diminution), MRI was used to confirm the diagnosis. Pericapsular edema, effusion around the biceps tendon, obliteration of the fat in the subcoracoid triangle, and thickness of the anterior band of the inferior glenohumeral ligament were the main criteria used to confirm the diagnosis.^25^

### Statistical Analysis

Statistical analysis used the Data Analysis package in Microsoft Excel (Microsoft, Richmond, WA). To verify normal distribution, the Kolmogorov-Smirnov test was applied. Descriptive statistics for quantitative variables included mean and SD. The Student *t* test was used for variables with a normal distribution, whereas the Mann-Whitney *U* test was applied for those without a normal distribution. Odds ratios (ORs) were calculated for surgery on the dominant side and smoking status to evaluate their impact on repair failure and the occurrence of adhesive capsulitis. The level of statistical significance was defined as *P* < .05.

## Results

A total of 63 patients, 4 of whom presented with bilateral injuries, were treated with suture bridge rotator cuff repair and were prospectively enrolled in this study (67 shoulders). At the time of surgery, all patients were younger than 40 years; all attended the follow-up consultations. The average follow-up period was 66.8 months (range, 62.4-88.6 months). The mean age at the time of surgery was 33.6 years (range, 21-40 years). Of the patients, 8 (13%) were professional athletes, 39 (62%) occasionally participated in sports, and 16 (25%) reported no sportive activity (Table 1). In 36 shoulders (54%), only the supraspinatus was torn. In 29 patients (43%), the supraspinatus and infraspinatus tendons were torn. There were 56 full-thickness lesions (84%) and 11 partial tears (16%). The latter were Ellman grade 3—considered PASTA (partial articular supraspinatus tendon avulsion) lesions—in 8 shoulders (73%) and Ellman grade 2 in 3 shoulders (27%) (Table 1). Concomitant all-arthroscopic procedures included 55 subacromial decompressions, 9 distal clavicle resections, and 13 cases of tenotomy and suprapectoral tenodesis of the long head of the biceps for all patients presenting lesions at this level.

**Table 1:** Demographic and Lesion Characteristics

Parameter	Data (67 Shoulders)
Mean age at surgery (range), yr	33.6 (21-40)
Sex: male/female	35 (55.5)/28 (44.5)
Professional activity: manual labor	54 (86)
Surgery on dominant side	52 (78)
Professional athlete	8 (13)
Occasional sports	39 (62)
No sportive activity	16 (25)
Average follow-up (range), mo	66.8 (62.4-88.6)
Tear location	
Supraspinatus	36 (54)
Supraspinatus + infraspinatus	29 (43)
Supraspinatus + infraspinatus + teres minor	1 (1.5)
Infraspinatus + teres minor	1 (1.5)
Full-thickness lesion	56 (84)
Partial tear (Ellman classification)	11 (16)
Grade 2	3 (27)
Grade 3	8 (73)
Tendon retraction (Patte classification)	24 (21)
Type 1	12 (18)
Type 2	9 (13.4)
Type 3	3 (4.5)
Fatty infiltration (Goutallier-Bernageau classification)	13 (19)
Stage 1	6 (9)
Stage 2	7 (10)

NOTE. Data are presented number (percentage) unless otherwise indicated.

At final follow-up, all clinical outcome scores improved significantly after suture bridge repair. The mean Constant score increased significantly from 39.8 points (range, 29-52 points) to 88.9 points (range, 34-100 points) postoperatively (*P* < .001). The average difference in the Constant score postoperatively was 49.1 points (SD, 17.1 points). The difference between preoperative and postoperative scores was greater than the MCID for 92.54% of shoulders. Mean forward anterior flexion improved from 86° (range, 60°-110°) to 137° (range, 90°-180°) postoperatively (*P* < .001). A significant difference in objective muscular strength score was observed, increasing from 10.8 points (range, 0-16 points) preoperatively to 19.5 points (range, 10-25 points) postoperatively (*P* < .001). Subjectively, the patients appreciate the force, postoperatively, at 91% (range, 30%-100%) compared with the contralateral side (Table 2).

**Table 2:** Functional Outcomes

Item	Preoperatively	Postoperatively	Statistical Significance
Constant score, points	39.8 (29-52)	88.9 (34-100)	*P* < .001
ASES score, points	41.8 (30-64)	90.2 (35-100)	*P* < .001
Forward flexion, °	86 (60-100)	137 (90-180)	*P* < .001
Abduction, °	66 (15-95)	134 (80-150)	*P* < .001
Strength, points	10.8 (0-16)	19.6 (10-25)	*P* < .001
External rotation	Hand above head and elbow forward	Full elevation of arm	*P* < .05
Internal rotation	L3 (buttock-T12)	T8 (sacrum-T12)	*P* < .001
VAS score	6.3 (3-10)	1.3 (0-9)	*P* < .001

NOTE. Data are presented as mean (range).

ASES, American Shoulder and Elbow Surgeons; VAS, visual analog scale.

Pain significantly decreased after surgery, with the VAS score descending from 6.3 (range, 3-10) to 1.3 (range, 0-9) postoperatively (*P* < .001). The ASES score improved significantly from 41.8 (range, 30-64) preoperatively to 90.2 (range, 35-100) postoperatively (*P* < .001). The average difference in the ASES score postoperatively was 48.3 points (SD, 17 points). The difference between preoperative and postoperative scores was greater than the MCID for 88.24% of shoulders. After surgery, 53 patients (84%) returned to work at an average of 10.1 months (range, 6-12 months) later. Among the included patients, 33 (85%) who engaged in occasional sports activities successfully resumed their previous activity levels after 9.8 months (range, 6-12 months); 6 elite athletes (67%) similarly returned to their preinjury levels of sports after 10.7 months (range, 9-12 months), whereas 2 (22%) returned to an inferior level. At final follow-up, 95% of patients were satisfied or very satisfied (Table 2). No statistically significant difference in the outcomes was found between patients with full-thickness tears and those with partial-thickness tears (Table 3).

**Table 3:** Comparison of Outcomes Between Full- and Partial-Thickness Tears

Outcomes	Full-Thickness Tear	Partial-Thickness Tear	Statistical Significance
Constant score, points	88.6 (34-100)	90.3 (63-100)	*P* = .75
ASES score, points	90.1 (35-100)	90.7 (65-100)	*P* = .9
Forward flexion, °	136 (90-180)	140 (120-170)	*P* = .63
Abduction, °	134 (80-150)	136 (90-150)	*P* = .72
Strength, points	19.3 (10-25)	20.8 (11-25)	*P* = .25
External rotation	Full elevation of arm	Full elevation of arm	*P* = .73
Internal rotation	T8	T8	*P* = .96
VAS score	1.4 (0-9)	0.5 (0-5)	*P* = .19

NOTE. Data are presented as mean (range).

ASES, American Shoulder and Elbow Surgeons; VAS, visual analog scale.

### Assessment of Tendon Repair Healing

**Fig 3:**
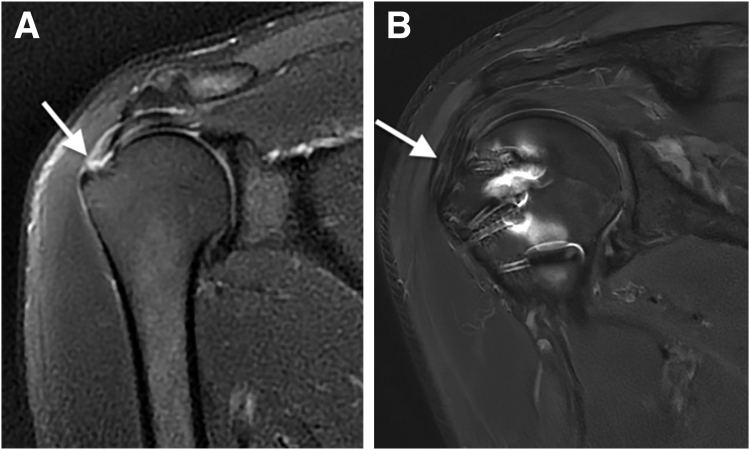
Magnetic resonance imaging (T2 fat-supression frontal view) of right shoulder showing full-thickness tear of supraspinatus tendon (arrow) (A) and healed supraspinatus tendon (arrow) (Sugaya stage 1) at 12 months after arthroscopic repair (B).

**Fig 4:**
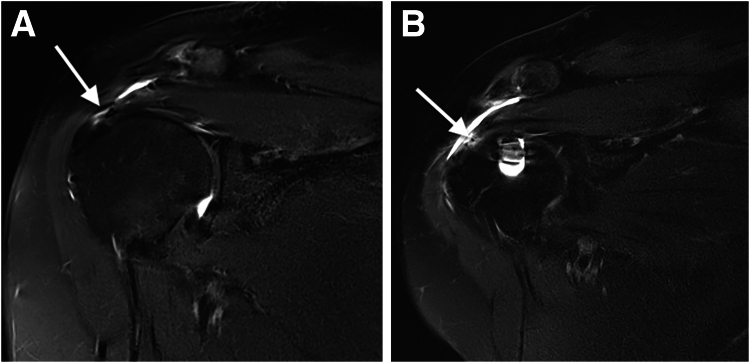
Magnetic resonance imaging (T2 fat-supression frontal view) of right shoulder showing full-thickness tear of supraspinatus (arrow) (A) and minor discontinuity (arrow) suggesting small full-thickness tear (Sugaya stage 4) at 12 months after arthroscopic repair (B).

According to the Sugaya classification on MRI, 60 rotators cuffs (90%) were considered healed (stage 1 in 47 and stage 2 in 13) at 12 months after surgery (Fig 3). Rerupture of the repaired cuff was observed in 2 patients. Failure of surgical repair was observed in 5 patients (7%); this was defined as Sugaya stage 3 in 4 patients (6%) and stage 4 in 1 patient (1.5%) (Fig 4). Diagnosis of failure was made after the systematic MRI evaluation at 12 months. At final follow-up, the failure rate—encompassing both tendon nonhealing and rerupture—was calculated to be 10%. All the patients presenting with traumatic rerupture of the repaired rotator cuff were surgically treated using the same arthroscopic technique, followed by complete healing at final follow-up.

A lower mean Constant score was observed in patients presenting with nonhealing of tendons after surgery (83.7 points; range, 35-97 points) than in those whose tendons were considered healed (Sugaya stages 1 and 2) (88.6 points; range, 34-100 points); however, the difference was not statistically significant (*P* = .47). The average pain level was 2.4 in the group with unhealed cuffs versus 1.2 in the healed group (Sugaya stages 1 and 2) (*P* = .2). Surgery on the dominant side presented an OR of 7.38 (95% confidence interval [CI], 0.85-63.5; *P* = .06) for repair failure, whereas smoking status was associated with an OR of 2.14 (95% CI, 0.43-10.46; *P* = .34).

### Complications

Five cases of adhesive capsulitis were diagnosed and confirmed by MRI. Smokers presented with an OR of 15.07 (95% CI, 1.54-146.67; *P* = .01) for this complication. All patients presenting with adhesive capsulitis had favorable results at final follow-up after intra-articular infiltration in addition to physical therapy. No infection was noted in the included patients.

## Discussion

The principal findings of this study on arthroscopic rotator cuff repair using a suture bridge technique in patients under 40 years old showed significant improvements in clinical scores (Constant and ASES scores), pain levels, and patient satisfaction, highlighting the importance of this technique for this particular population. During follow-up, Constant scores improved after arthroscopic rotator cuff repair with the suture bridge technique. Forward flexion increased by 51°, muscular strength increased by 8.7 points, and patients rated their strength at 91% of that of the contralateral shoulder at final follow-up.

The existing studies are retrospective and have presented variable clinical and imaging follow-up periods, ranging from 13 to 48 months. Moreover, no study has evaluated the arthroscopic double-row suture bridge repair technique individually in patients under 40 years old (Table 4).^26^, ^27^, ^28^, ^29^, ^30^, ^31^

**Table 4:** Findings of Previous Studies Examining Arthroscopic Rotator Cuff Repairs in Young Population

Authors	Level of Evidence	Mean Age, yr	Patients, N	Mean Follow-Up, mo	Technique	Functional Results at Last Follow-Up
Lin et al.^26^ (2013)	III	37.5 (range, 16.2-44.9)	53	35.8 (range, 13.8-59.1)	Single and double row	Mean ASES score: 84.6 (range, 21.6-100.0) Mean forward flexion: 168.4° (range, 120°-180°)
Tambe et al.^27^ (2009)	III	25.7 (range, 19-31)	11	18 (range, 12-31)	Single and double row	Mean Constant score: 99 (range, 92-100) Mean Oxford Shoulder Score: 18 (range, 4-16)
Krishnan et al.^28^ (2008)	III	37 (range, 21-39)	23	26 (range, 24-29)	Single row	Mean ASES score: 92 (range, 65-100) Mean active anterior elevation: 160° (range, 155°-170°) Mean active external rotation: 60° (range, 50°-85°) Mean internal rotation: T12 (range, T2-L2)
Reynolds et al.^29^ (2008)	III	25.6 (SD, 5.5)	82	38 (range, 18-59)	Arthroscopic debridement	ASORS score > 60: 76% Return to same level of sport: 54% No return to sport: 24%
Payne et al.^30^ (1997)	III	28 (range, 25-33)	43	48 (range, 24-120)	Arthroscopic debridement	Overall success rate: 72% Superior ASES score after surgery in cases of acute trauma compared with chronic lesions
Andrews et al.^31^ (1985)	III	22 (range, 16-29)	36	13.1 (SD, NR)	Arthroscopic debridement	Good or excellent results: 85% Poor results: 15%

ASES, American Shoulder and Elbow Surgeons; ASORS, Athletic Shoulder Outcome Rating Scale; NR, not reported; SD, standard deviation.

In the study of Lin et al.^26^ including 53 patients, forward flexion (mean, 168.4°; range, 120°-180°) and the ASES score (mean, 84.6 points; range, 21.6-100.0 points) improved significantly after arthroscopic repair of RCTs. A mean ASES score of 92 points and forward elevation and external rotation measurements of 170° and 60°, respectively, were reported by Krishnan et al.^28^

Postoperatively, our study showed a significant reduction in pain levels, as indicated by the VAS score, with scores decreasing from an initial average of 6.3 (range, 3-10) to a postoperative average of 1.3 (range, 0-9) (*P* < .001). A few studies have examined the link between age and the results of arthroscopic rotator cuff repairs. Cole et al.^32^ compared the results of patients aged under 50 years with those of patients aged between 50 and 59 years, aged between 60 and 69 years, and aged 70 years or older. The VAS score and external rotation strength were found to be significantly improved in the group aged under 50 years compared with those aged 60 to 69 years. In the study of Milano et al.,^33^ a negative correlation was found between age and Constant score (*P* = 0.244).

In our study, supraspinatus tendon tears were reported in 54% of operated shoulders. Azzam et al.^34^ examined 32 teenagers (28 boys and 4 girls) with a mean age of 16.1 years who received surgical treatment for RCTs. Traumatic etiology was found in 91% of cases (29 patients), with 27 of these patients (93%) having no symptoms prior to the incident. The most encountered tear was a supraspinatus lesion, present in 21 patients (66%). Of the patients, 14 (56%) were treated with a single-row repair, with the rest (44%) receiving a double-row repair. At a mean follow-up of 6.2 years (range, 2-10 years), the mean ASES score was 93 points, the mean Western Ontario Rotator Cuff score was 89%, and the mean numeric pain rating was 0.3.

In our study, surgical repair of RCTs in the young adult population resulted in excellent functional outcomes at mid-term follow-up. Dilisio et al.^35^ reported a return to sport with mild or no shoulder pain in all 9 patients included in their study. A meta-analysis performed by Klouche et al.^36^ examined 25 studies comprising 859 patients to evaluate return to play after surgical management of RCTs. They noted an 84.7% return-to-play rate, with 65.9% of patients returning to the preinjury level. However, only 49.9% of professional athletes participated at the same level after their injury.^36^ In our cohort, 67% of elite athletes returned to the preinjury level of sport. Mazoué and Andrews^37^ studied a cohort of 16 professional baseball players who underwent repair of full-thickness RCTs. In their series, they concluded that it is difficult for baseball players to return to an elite level of play after these types of injuries given that only 1 pitcher (out of 12) and 1 of 2 position players were able to return to professional baseball after surgery performed on the dominant arm. After the surgical intervention, 84% of our patients resumed work within an average duration of 10.1 months (range, 6-12 months). This could be attributed to the biomechanical properties of the applied construct allowing early mobilization. This result is of importance in this demanding population because it indicates a satisfactory high rate of return to the preinjury level of activity. Krishnan et al.^28^ reported an excellent return to preinjury functional levels (90%) and a return-to-work rate of 90%. Few studies have reported on the clinical results of RCTs in athletic and, therefore, younger patients.

Among the patients included in our study, 95% were satisfied or very satisfied at final follow-up. Lin et al.^26^ reported a 96.2% satisfaction rate at final follow-up after primary arthroscopic repair of full-thickness RCTs in young patients. Hawkins et al.^19^ found a satisfaction rate of 68% in a series of patients aged 40 years or younger who had full-thickness tears treated by open surgery.

Miyazaki et al.^38^ analyzed the results of the aforementioned technique in a population with a mean age of 60 years and mean follow-up period of 30 months. Thirty-seven patients were included in the study and had a mean final University of California, Los Angeles score of 33.7, with a failure rate of 2.7%. Lee et al.^39^ examined the results of 2 different techniques, speed bridge (SB) and modified tension band, in 141 patients with an average age of 59 years presenting with full-thickness supraspinatus tears. At last follow-up, the mean Constant and ASES scores were 85 points and 88.2 points, respectively, in the SB group and 86 points and 89 points, respectively, in the modified tension band group. However, Lee et al. observed a higher failure rate in the SB group compared with the results we obtained. These results could be influenced by the older age of the included patients.^39^

The excellent functional outcomes in this study were substantiated by MRI findings. According to the Sugaya classification of tendon healing after surgery, 90% of rotator cuff repairs were categorized as presenting with a Sugaya stage 1 or 2 rotator cuff, thus indicating successful healing. In a retrospective multicenter study, Werthel et al.^40^ compared the functional outcomes and osteoarthritis rates of tendons classified as Sugaya stage 3 in a cohort of 203 shoulders. The average age was 51 ± 8 years, and the average follow-up period was 11.5 years. The mean Constant scores and average strength values were significantly higher in Sugaya stage 1 and 2 tendons compared with Sugaya stage 3 tendons (*P* = .021 and *P* = .003, respectively), as well as Sugaya stage 4 and 5 tendons (*P* = .07 and *P* = .038, respectively), but did not differ between Sugaya stage 3 tendons and Sugaya stage 4 and 5 tendons. The mean Subjective Shoulder Value and acromiohumeral index were significantly higher. Fatty infiltration, pain, and progression of osteoarthritis according to the Hamada classification were significantly lower in Sugaya stage 1 and 2 tendons and Sugaya stage 3 tendons compared with Sugaya stage 4 and 5 tendons (*P* < .05), with no significant difference between Sugaya stage 1 and 2 tendons and Sugaya stage 3 tendons.^40^ Boileau et al.^41^ examined rotator cuffs using MRI or computed tomographic arthrography and found that age negatively influenced tendon healing: Healing was observed in 95% of patients younger than 55 years compared with 58% of those aged 55 years or older.

A few studies have reported on long-term follow-up of the double-row suture bridge technique. Dukan et al.^13^ reported on the results of 68 patients treated for chronic nontraumatic supraspinatus tears using an arthroscopic double-row technique. This prospective study had a mean follow-up period of 68 months. At latest follow-up, the mean ASES and Constant scores improved from 48.2 to 87.4 points and from 37.8 to 82.8 points, respectively. Control MRI showed a healing rate of 88% (Sugaya stage 1-2).^13^

These superior results could be attributed to the biomechanical properties of the suture bridge repair combined with braided suture tapes.^42^ This technique allows increased load resistance and pressure enhancement at the bone-tendon interface. Contact area and coverage are also increased. This conformation allows a better distribution of the loads, especially in rotation, in which the repair is most fragile. This is clinically relevant because it permits early mobilization and, therefore, decreases the risk of postoperative stiffness and could result in good functional outcomes.^16^^,^^33^

In addition, the use of suture tapes enhances the apposition of the tendon on the footprint.^33^ The even distribution of tension decreases the risk of the sutures cutting through on the medial part of the construct, thus possibly explaining the lack of failures in this particular area in our repairs.^16^^,^^43^

### Limitations

This study presents several limitations. First, it was not randomized; it included a series of consecutive patients operated on at a single center by a single surgeon. Furthermore, the postoperative rehabilitation protocol and MRI techniques may not have been standardized, which limits external validity. Second, the control MRI scan was performed at the 1-year follow-up, which may lead to underestimation of the retear rate. Third, the suture bridge technique was performed for all tear types, and this could represent a limitation factor because different types of lesions could be suitable for other types of repair techniques. Another source of confounding bias could be a result of additional procedures performed during surgery in certain patients, such as acromioplasty, tenotomy, and tenodesis of the long head of the biceps, given that these surgical procedures can lead to pain relief and improvements in range of motion.

## Conclusions

The use of a suture bridge technique in arthroscopic RCT repair for patients under 40 years of age resulted in excellent long-term outcomes, including successful tendon healing, pain relief, improved shoulder function, and high patient satisfaction. This result is of significant importance in this demanding population because it allows a satisfactory high rate of return to the preinjury level of activity.

## Disclosures

All authors (L.C.F., F.A., M-A.R., P.B.) declare that they have no known competing financial interests or personal relationships that could have appeared to influence the work reported in this paper.
